# Navigation-Assisted Ventriculoperitoneal Shunt Placement in Pediatric Hydrocephalus: Improved Catheter Positioning and Reduced Revision Rates

**DOI:** 10.3390/medicina62030424

**Published:** 2026-02-24

**Authors:** Emrullah Cem Kesilmez, Muharrem Furkan Yüzbaşı, Muhammed Kırkgeçit, Hasan Türkoğlu, Kasım Zafer Yüksel

**Affiliations:** 1 Neurosurgery Clinic, Kahramanmaraş Sütçü İmam University, Kahramanmaraş 46050, Türkiye; 2 Neurosurgery Clinic, MegaPoint Hospital, Kahramanmaraş 46100, Türkiye; 3 Neurosurgery Clinic, Gaziantep City Hospital, Gaziantep 27470, Türkiye

**Keywords:** hydrocephalus surgery, ventriculoperitoneal shunt, neuronavigation surgery, computer-assisted, child, treatment outcome, reoperation

## Abstract

*Objective*: This study aimed to compare the clinical outcomes of navigation-assisted and conventional (freehand) ventriculoperitoneal (VP) shunt placement in pediatric hydrocephalus patients. *Methods*: A retrospective review was conducted of 164 patients under the age of 18 who underwent VP shunt placement for hydrocephalus between 2015 and 2023 and had a minimum postoperative follow-up of 12 months. The conventional technique was used in 116 patients. The navigation-assisted technique (intraoperative ultrasonography or frameless neuronavigation) was used in 48 patients. Demographic data, hydrocephalus etiology, catheter tip position (Yim classification), revision rates, infection, complications, and length of hospital stay were recorded. Catheter tip position was assessed on postoperative imaging by two independent investigators. *Results*: No significant differences were found between the groups in terms of age, sex, and hydrocephalus etiology. The optimal catheter placement rate was significantly higher in the navigation-assisted group compared to the conventional technique (81.25% vs. 60.34%, *p* = 0.017). The revision rate was significantly lower in the navigation-assisted group (16.67% vs. 38.79%, *p* = 0.010). The mean hospital stay was shorter in the navigation-assisted group (7.85 ± 3.97 days vs. 10.20 ± 3.70 days, *p* < 0.001). The groups were similar in terms of infection (2.08% vs. 9.48%, *p* = 0.183) and overall complication rates (14.58% vs. 16.38%, *p* = 0.959). *Conclusions*: Navigation-assisted VP shunt placement in pediatric hydrocephalus patients is associated with a high rate of optimal catheter position, a low revision rate, and a short hospital stay. These findings support the use of navigation technology in pediatric hydrocephalus surgery, but also reveal that infection and complications are unassociated with the surgical technique.

## 1. Introduction

Hydrocephalus causes ventricular enlargement due to impaired cerebrospinal fluid (CSF) circulation and leads to neurodevelopmental problems in children. Treatment is surgical and the primary objective is to divert CSF from the ventricles to the peritoneal cavity, which is most commonly achieved by ventriculoperitoneal (VP) shunts [1]. However, shunts carry a lifelong risk of complications. Population-based longitudinal data indicate a 30–40% need for revision within the first year, while more than half of the patients require revision in the long term, with the majority occurring in the first decade of life [2,3]. Revisions are risky for the patient and can strain the healthcare system, and therefore, initial success and safety are crucial [4].

The classical (freehand) approach involves catheter placement based on cranial surface anatomy and external landmarks; however, in infants, conditions such as narrow ventricles, midline shift, and fontanelle closure have been associated with malposition patterns such as catheter tip contact with the wall or choroid plexus, or parenchymal placement [5]. Malposition may increase the risk of proximal obstruction, inadequate CSF drainage, and early loss of function [2]. Therefore, objective and reproducible assessment of catheter positioning is crucial. Yim’s four-level scale, frequently used in clinical research, categorizes placement within the ipsilateral frontal horn and Monro periphery as “optimal,” and placement in the contralateral horn, non-target CSF space (e.g., third ventricle), and parenchymal locations as increasingly suboptimal [6].

The accuracy of placement has increased with the use of image-guided approaches. Comparative series and systematic reviews report that ventricular catheter placement guided by ultrasound and/or stereotactic navigation significantly improves positioning accuracy compared with the freehand technique and may reduce the risk of malposition and proximal shunt failure [7,8]. Although some researchers suggest no difference in short-term functional outcomes relative to freehand placement [9], current trends indicate that outcomes are improving with the multicomponent approaches, more widespread use of antibiotic-impregnated catheters, and image-guided placement [10].

The majority of studies on this topic in the literature are reported from high-income countries, while large series comparing long-term outcomes between freehand and navigation-assisted techniques in pediatric populations from middle-income countries like Turkey are limited. Furthermore, most existing studies focus on optimal catheter placement, but there are few studies that have comprehensively evaluated the relationship between radiological features and clinical outcomes such as revision rate, length of hospital stay, and complications. In this context, presenting large series demonstrating the long-term comparative outcomes of freehand and navigation-assisted VP shunt placement in pediatric cases in our country is important both to strengthen local standards and to contribute to the global literature. As such, we aimed to compare freehand and image-guided approaches in terms of optimal catheter position, revision rate, and infection outcomes in patients under 18 years of age who underwent surgery at a single center between 2015 and 2023.

## 2. Materials and Methods

This study was conducted in pediatric patients who underwent VP shunt placement due to hydrocephalus between January 2015 and December 2023 at the Department of Brain and Neurosurgery, Kahramanmaraş Sütçü İmam University Faculty of Medicine. The study of this cohort and all processes of the study were approved by the Kahramanmaraş Sütçü İmam University Medical Research Ethics Committee (Session No: 2024/05 Decision No: 22). All procedures were performed in accordance with the principles of the Declaration of Helsinki.

### 2.1. Study Group

The study included patients under the age of 18 who underwent initial VP shunt placement due to hydrocephalus and who had at least 12 months of postoperative follow-up data. A total of 164 patients met the study criteria. Among these, 116 had undergone shunt placement using the conventional (freehand) technique and 48 underwent navigation-assisted shunt placement. Patients who underwent only distal or proximal shunt revision, those who underwent ventriculoatrial or pleural shunting, those who underwent only endoscopic third ventriculostomy (ETV), patients with a follow-up shorter than 12 months, and those with incomplete or unavailable medical records were excluded from the study.

### 2.2. Clinical Data and Measurements

Patient demographics, hydrocephalus etiology, preoperative neurological status, and comorbidities were recorded from patient files. Postoperative computed tomography (CT) and/or magnetic resonance imaging (MRI) scans were independently evaluated by two investigators blinded to the study, and ventricular catheter tip position was graded according to the Yim classification:

- Grade I (optimal): Catheter tip in free CSF within the ipsilateral frontal horn or near the foramen of Monro, without contact with the ventricular wall or septum;
- Grade II (acceptable): Catheter tip in the contralateral frontal horn;
- Grade III (suboptimal): Catheter tip in a non-target CSF space (e.g., third ventricle);
- Grade IV (malposition): Catheter tip within brain parenchyma [6]. In case of inter-rater disagreement, a third experienced neurosurgeon was consulted.

Complications were evaluated during the 12-month postoperative follow-up period. Shunt infection was confirmed by clinical findings (fever, wound discharge, signs of meningitis) combined with microbiological confirmation (CSF culture positivity). Bleeding was defined as newly developed intraventricular or parenchymal hemorrhage on postoperative CT. Subdural collection was defined as subdural effusion or hematoma detected radiologically and presenting clinical findings. Excessive drainage was defined as excessive CSF drainage manifesting as headache, subdural collection, or slit ventricle syndrome.

The need for revision was determined by the presence of mechanical obstruction (catheter blockage), shunt infection, catheter malposition, clinical deterioration due to shunt dysfunction, radiological progression, or abdominal complications (cyst, peritonitis). Hospital stay was recorded in days from the day of surgery to the day of discharge.

### 2.3. Surgical Details

Patients were not randomly assigned to surgical groups. While the conventional technique was routinely used in the early stages of the study (2015–2018), the navigation-assisted technique was implemented in selected cases after the navigation system became available in our clinic (as of 2019). The choice of technique was determined by the surgeon’s judgment, patient age, ventricular anatomy, and other factors deemed relevant on a case-by-case basis.

All patients underwent preoperative neuroimaging. Routine CT scans were performed in the conventional technique group, while a thin-section MRI protocol was performed in the navigation-assisted group. Intravenous cefazolin (30–50 mg/kg, maximum 2 g) was administered as a prophylactic antibiotic 30 min before surgery. All procedures were performed under general anesthesia. Patients were immobilized in a supine position, with their heads turned approximately 45 degrees laterally from a neutral position.

***Freehand Technique*:** The ventricular catheter insertion point was Kocher’s point (1–2 cm anterior to the coronal suture and 2–3 cm lateral to the midline) for the frontal approach or Keen’s point for the parietal approach. The insertion point was determined based on the surgeon’s preference, ventricular anatomy, and the type of hydrocephalus. After a skin incision, a cranial bur hole (10–14 mm) was created, and the dura was coagulated with cautery. Anatomical landmarks were used as a guide for ventricular catheter insertion. In the frontal approach, the catheter was advanced toward the medial canthus, parallel to the tragus in the cranial plane, and toward the midpoint of the pupil in the coronal plane. The target depth was approximately 4–5 cm for young children and 3–4 cm for infants. Cannulation of the ventricle was confirmed by the disappearance of resistance and observation of free CSF flow as the catheter entered the ventricle. After adequate CSF flow was achieved, the stylet was removed, and the proximal end of the catheter was connected to the valve of the shunt system.

***Navigation-Assisted Technique:*** In navigation-assisted cases, preoperative images were uploaded to the frameless neuronavigation system. After the patient was placed on the operating table, registration was performed using a reference frame, and the registration error was kept below 2 mm. Three-dimensional reconstruction was used on the navigation system screen to visualize the ventricular anatomy, and the optimal catheter insertion point and trajectory were determined. In cases where intraoperative ultrasonography was used, especially in infants with a patent fontanelle, a probe was placed through the dura after the bur hole was made, and ventricular location and dimensions were assessed in real-time. The optimal insertion point was marked under navigation. After the bur hole was opened, the ventricular catheter was advanced toward the anterior ipsilateral frontal horn or the level of the foramen of Monro, in line with the optimal trajectory visualized on the navigation system. As the catheter was advanced, its position was monitored in real-time on the navigation screen, and when the target point was reached, CSF flow was checked to confirm the optimal position.

Within the navigation-assisted group, intraoperative ultrasonography alone was used in 18 patients (37.5%), frameless neuronavigation alone in 20 patients (41.7%), and both modalities combined in 10 patients (20.8%). Ultrasonography was preferred in younger patients with patent fontanelles, while frameless neuronavigation was selected for older children with closed fontanelles, narrow ventricles, or complex anatomy.

***Shunt System and Wound Closure:*** In both techniques, the proximal catheter was connected to the valve, and the shunt system was advanced through subgaleal and subcutaneous tunnels, via the cervical and thoracic regions, to the upper abdominal quadrant. The distal catheter was inserted into the peritoneal cavity through a small transverse or paramedian abdominal incision (approximately 2–3 cm) and left for a sufficient length (approximately 30–40 cm) within the peritoneum. In cases where programmable valves were used, valve pressure was adjusted individually based on the patient’s age, ventricular size, and the etiology of hydrocephalus. All incisions were closed in layers with absorbable sutures. Patients were evaluated with a cranial CT scan within the first 24–48 h after surgery to check catheter position and potential complications.

### 2.4. Follow-Up Details

Patients were followed up at regular intervals in the outpatient clinic during the postoperative period. Clinical examination, wound control, and neurological assessment were performed at 1, 2, and 4 weeks postoperatively during the first month, and CT or ultrasonography were repeated in cases of doubt. Head circumference measurement, neurological examination, and developmental assessment were performed at monthly outpatient clinic visits between 1 and 12 months, and cranial imaging (CT or MRI) was repeated at 3, 6, and 12 months. After 12 months, asymptomatic patients were followed at 6-month intervals, and in symptomatic patients (vomiting, lethargy, irritability, headache, fontanel tension), urgent imaging was performed to investigate shunt dysfunction. In patients requiring revision, the reason for revision, timing, and postoperative course were recorded in detail, and revision-free survival time was calculated.

### 2.5. Statistical Analysis

All analyses were conducted using IBM SPSS version 27.0 (IBM Corp., Armonk, NY, USA). *p*-values less than 0.05 were accepted as statistically significant. Histogram and Q-Q plots were used to determine whether continuous variables are normally distributed. Descriptive statistics were presented using mean ± standard deviation for normally distributed continuous variables, median (25th percentile–75th percentile) for non-normally distributed continuous variables and frequency (percentage) for categorical variables. Continuous variables were analyzed using Student’s *t* test or the Mann–Whitney U test depending normality of distribution. Categorical variables were analyzed using the chi-square test or Fisher’s exact test or Fisher–Freeman–Halton test. Univariable and multivariable (adjusted by age and sex) logistic and linear regression analyses were performed to evaluate relationships between navigation-assisted surgery and outcomes.

## 3. Results

The groups consisted of 116 (70.7%) patients who underwent the freehand technique and 48 (29.3%) who underwent the navigation-assisted technique. No significant difference was found between the groups in terms of age, gender, or hydrocephalus etiology (Table 1).

**Figure 1:**
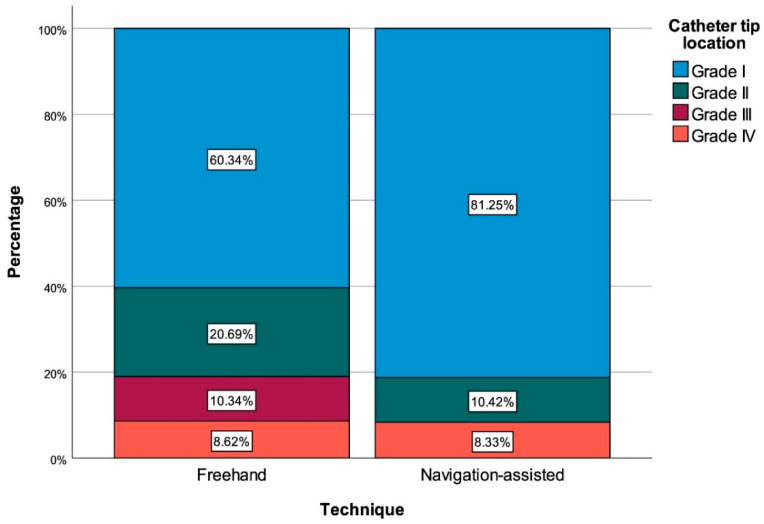
Catheter tip location with regard to surgical technique.

**Figure 2:**
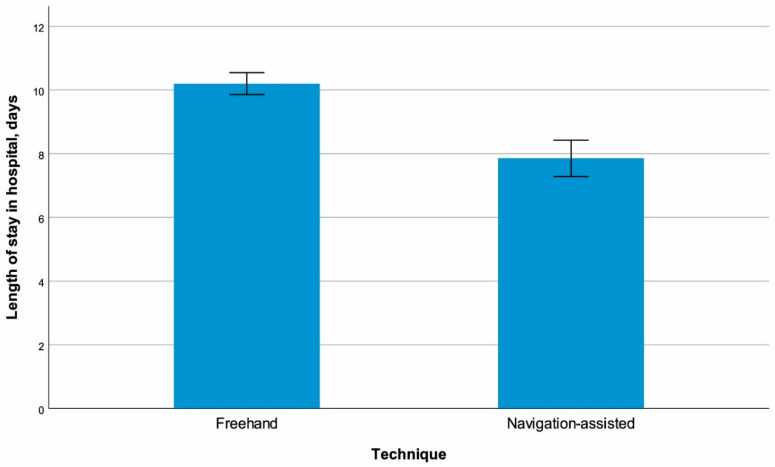
Length of stay in hospital with regard to surgical technique.

**Figure 3:**
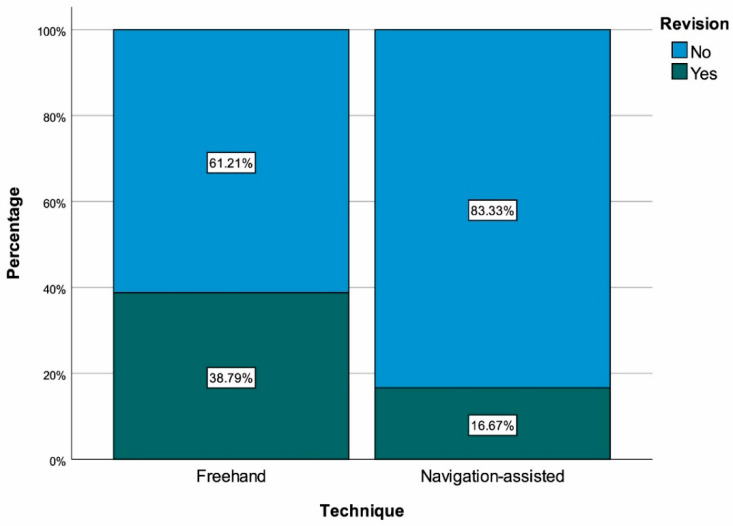
Revision with regard to surgical technique.

The frequency of optimal catheter placement (Grade I) was significantly higher in the navigation-assisted group than in the conventional technique group (81.25% vs. 60.34%, *p* = 0.017) (Figure 1). Hospital stay was significantly shorter in the navigation-assisted group (7.85 ± 3.97 days vs. 10.20 ± 3.70 days, *p* < 0.001) (Figure 2). The revision rate was significantly lower in the navigation-assisted group (16.67% vs. 38.79%, *p* = 0.010) (Figure 3). No significant difference was found between the groups in terms of infection and general complication rates (*p* = 0.183 and *p* = 0.959, respectively) (Table 1).

Exploratory analysis within the navigation-assisted cohort showed Grade I placement rates of 77.8% (14/18) for ultrasonography, 85.0% (17/20) for frameless neuronavigation, and 80.0% (8/10) for combined use; formal comparison was not performed due to small subgroup sizes. The low infection event rate (*n* = 1 in navigation group) resulted in wide confidence intervals, indicating substantial statistical uncertainty for this outcome.

Navigation-assisted surgery was independently associated with increased chance of grade I catheter tip location (OR: 2.839, 95% CI: 1.241–6.496, *p* = 0.013), decreased length of stay in hospital (b: −2.357, 95% CI: −3.631–−1.083, *p* < 0.001) and decreased risk of revision (OR: 0.312, 95% CI: 0.134–0.728, *p* = 0.007) after adjustment for age and sex. On the other hand, infection (OR: 0.192, 95% CI: 0.024–1.547, *p* = 0.121) and complication (OR: 0.874, 95% CI: 0.339–2.254, *p* = 0.780) were not independently associated with type of surgery after being adjusted for age and sex (Table 2).

## 4. Discussion

The present study demonstrates that image-guided VP shunt placement significantly improves catheter positioning accuracy compared with freehand technique in pediatric patients. This finding aligns with recent evidence supporting navigation technology in neurosurgery. Stieglitz et al. [11] reported a mean deviation of catheter tips from planned position of only 1.5 mm using frameless stereotaxy, while Besharati Tabrizi and Mahvash [12] demonstrated submillimetric accuracy (0.8 ± 0.25 mm projection error) with augmented reality–guided neurosurgery. Recent advances continue to refine these technologies; Carbone et al. [13] showed that novel AR head-mounted displays achieved 2 mm median targeting accuracy, comparable to commercial optical tracking systems. Wilson et al. [5] demonstrated that both stereotactic and ultrasound-guided placements were significantly more accurate than freehand technique (*p* < 0.001), with optimal placement rates exceeding 85% in image-guided cases. Our optimal placement rate of 81.25% with navigation is consistent with these reports and with the meta-analysis by Nesvick et al. [14] (OR 2.31 for image guidance), supporting the reproducibility of this benefit across different settings. However, improvement in catheter accuracy does not uniformly translate to better shunt survival, as demonstrated by trials showing equivalent functional outcomes despite positioning differences.

Optimal catheter tip placement is critical for the long-term function of the shunt system and the need for revision. It is widely accepted in the literature that navigation-assisted techniques increase catheter placement accuracy [5,15]. In our study, the optimal catheter placement rate (Grade I) was significantly higher in the navigation-assisted group compared to the conventional technique (81.25% vs. 60.34%, *p* = 0.017). Series demonstrating that image-guided placement significantly improves catheter accuracy in pediatric patients, particularly those with narrow ventricles, support this finding [14]. However, there are also conflicting results in the literature. Factors such as catheter insertion site and trajectory may be more predictive of shunt survival than catheter tip position [16]. Additionally, improvement in catheter accuracy does not always increase shunt survival and that direct causality between structural accuracy and clinical outcome may not be established [8,17].

Hospital stay was significantly shorter in the navigation-assisted group (7.85 ± 3.97 days vs. 10.20 ± 3.70 days, *p* < 0.001). This difference likely reflects multiple factors: more accurate catheter positioning reducing early clinical concerns and prolonged observation periods, fewer index-hospitalization revisions, and more predictable CSF drainage facilitating earlier clinical stabilization [4]. However, temporal confounding must be considered. The 8-year study period (2015–2023) encompasses evolution in clinical practice, with navigation cases concentrated in the later years [10]. Although our surgical team remained constant throughout the study, with the same senior neurosurgeon leading all procedures and neuronavigation experience predating the study period (since 2013), and core protocols for antibiotic prophylaxis and shunt hardware remained unchanged, antibiotic-impregnated catheter use increased after 2019 and programmable valve selection evolved over time. These concurrent changes, rather than navigation technology alone, may partially account for improved outcomes in the later cohort [10]. In resource-limited healthcare systems like Turkey, reducing length of stay is critical for cost-effectiveness and reducing the socioeconomic burden on families, but the observed 2.3-day difference should be interpreted cautiously given potential era effects [4].

Shunt revision is one of the most important challenges in the management of pediatric hydrocephalus. In our study, the revision rate was significantly lower in the navigation-assisted group compared to the conventional technique (16.67% vs. 38.79%, *p* = 0.010). This finding is consistent with other comprehensive and/or multicenter studies demonstrating declines in shunt failure with navigation, excellent rates of optimal placement, and improved catheter safety in pediatric patients [18,19]. This association may reflect prevention of early mechanical failures, though the retrospective design precludes causal conclusions. The absolute risk reduction of 22.1% (NNT ≈ 4.5) is clinically meaningful but should be interpreted cautiously given non-randomized allocation and potential era effects. However, results showing non-superiority of navigation also exist. A 2022 systematic review reported that image guidance did not prolong shunt survival [8] and a trial reported that intraoperative ultrasound guidance did not significantly improve catheter placement accuracy [17]. These discrepancies suggest that effectiveness depends not only on the technology but also on factors such as the learning curve, patient selection, ventricular anatomy, and concurrent care improvements [8,17,20] Therefore, it is important to note that the observed reduction in revisions may be due to multifactorial improvements, not just navigation technology.

Infection rates did not differ significantly between groups (2.08% vs. 9.48%, *p* = 0.183), consistent with literature suggesting that infection control depends primarily on overall management protocols rather than catheter guidance method [21,22]. The numerically lower rate in the navigation group may reflect temporal confounding—increased antibiotic-impregnated catheter use after 2019 [23,24]—and the higher revision rate in the freehand group, as revision surgery independently increases infection risk [25]. Both groups showed predominantly early infections (≤30 days), suggesting perioperative factors drive infection risk [21,22]. However, with only one infection in the navigation group, our study was substantially underpowered for this outcome, and nonsignificant findings should not be interpreted as equivalence.

This study has several limitations. The non-randomized allocation and temporal clustering of techniques introduce selection and era bias that cannot be fully adjusted. Navigation was preferentially used for narrow ventricles and complex anatomy, potentially overestimating benefit in straightforward cases. Several variables were not systematically controlled: ventricular size was recorded categorically rather than quantitatively, entry site distribution differed between groups (frontal approach: ~75–80% freehand vs. ~85–90% navigation), and valve type was not included in regression models. The navigation-assisted group combined ultrasonography and frameless neuronavigation—techniques with different workflows and accuracy profiles—though subgroup analysis suggested comparable outcomes. The low infection event rate yields high type II error risk, and unmeasured factors including operative duration and case complexity may confound results. Finally, single-center findings may not generalize to other settings.

## 5. Conclusions

In this retrospective cohort, navigation-assisted VP shunt placement was associated with improved catheter positioning, reduced revision rates, and shorter hospital stays, while infection and complication rates did not differ between techniques. These findings support consideration of navigation technology as part of comprehensive pediatric hydrocephalus management, though the observational design precludes causal conclusions. Prospective randomized trials are needed to establish comparative effectiveness across navigation modalities and identify patients most likely to benefit.

## Figures and Tables

**Table 1:** Summary of variables with regard to surgical technique.

		Technique		
	Total (*n* = 164)	Freehand (*n* = 116)	Navigation-Assisted (*n* = 48)	*p*
Age, years	7 (2–13)	7 (3–12)	6.5 (2–15)	0.932 ^‡^
Sex				
Boy	70 (42.68%)	48 (41.38%)	22 (45.83%)	0.725 ^§^
Girl	94 (57.32%)	68 (58.62%)	26 (54.17%)	
Etiology				
Congenital	62 (37.80%)	47 (40.52%)	15 (31.25%)	0.476 ^§^
IVH	27 (16.46%)	17 (14.66%)	10 (20.83%)	
MMC	30 (18.29%)	19 (16.38%)	11 (22.92%)	
Tumor	45 (27.44%)	33 (28.45%)	12 (25.00%)	
Catheter tip location				
Grade I	109 (66.46%)	70 (60.34%)	39 (81.25%) *	**0.017 ^¶^**
Grade II	29 (17.68%)	24 (20.69%)	5 (10.42%)	
Grade III	12 (7.32%)	12 (10.34%)	0 (0.00%) *	
Grade IV	14 (8.54%)	10 (8.62%)	4 (8.33%)	
Length of stay in hospital, days	9.51 ± 3.92	10.20 ± 3.70	7.85 ± 3.97	**<0.001 ^†^**
Revision	53 (32.32%)	45 (38.79%)	8 (16.67%)	**0.010 ^§^**
Infection	12 (7.32%)	11 (9.48%)	1 (2.08%)	0.183 ^#^
Complication	26 (15.85%)	19 (16.38%)	7 (14.58%)	0.959 ^§^

Descriptive statistics are presented using mean ± standard deviation for normally distributed continuous variables, median (25th percentile–75th percentile) for non-normally distributed continuous variables and frequency (percentage) for categorical variables. † Student’s *t* test, ‡ Mann–Whitney U test, § Chi-square test, # Fisher’s exact test, ¶ Fisher–Freeman–Halton test, * Statistically significant category for the variables with three or more categories. Statistically significant *p* values are shown in bold.

**Table 2:** Unadjusted and adjusted relationships between navigation-assisted surgery and outcomes, regression analysis results.

	Unadjusted		Adjusted ^(1)^	
	Test Statistic (95% CI)	*p*	Test Statistic (95% CI)	*p*
Catheter tip location, Grade I ^†^	2.848 (1.261–6.431)	**0.012**	2.839 (1.241–6.496)	**0.013**
Length of stay in hospital, days ^‡^	−2.344 (−3.626–−1.063)	**<0.001**	−2.357 (−3.631–−1.083)	**<0.001**
Revision ^†^	0.316 (0.135–0.735)	**0.008**	0.312 (0.134–0.728)	**0.007**
Infection ^†^	0.203 (0.025–1.619)	0.132	0.192 (0.024–1.547)	0.121
Complication ^†^	0.872 (0.340–2.232)	0.775	0.874 (0.339–2.254)	0.780

(1) Adjusted by age and sex, CI: Confidence interval. † Test statistic was given as odds ratio and calculated using logistic regression analysis. ‡ Test statistic was given as unstandardized regression coefficient and calculated using linear regression analysis. Statistically significant *p* values are shown in bold.

## Data Availability

The data presented in this study are available on request from the corresponding author due to privacy protections associated with clinical records.
